# Performance investigations on data protection algorithms in generalized multi protocol label switched optical networks

**DOI:** 10.1038/s41598-022-26942-0

**Published:** 2023-01-09

**Authors:** Monika, Simranjit Singh, Amit Wason

**Affiliations:** 1 Punjabi University, Patiala, Punjab India; 2 Ambala College of Engineering and Applied Research, Devsthali, Ambala, Haryana India

**Keywords:** Engineering, Electrical and electronic engineering, Fibre optics and optical communications

## Abstract

In future generation networks, data protection is a crucial necessity particularly when information is sent on a system. Network protection has three objectives known as discretion, reliability and accessibility. The most common procedure exploited to attain this ambition is encryption. The Generalized Multi Protocol Label Switched (GMPLS) optical networks are designed to survive Internet Protocol’s (IPʹs) unreliable delivery. In this paper, GMPLS network is proposed with random users and then the data protection algorithms have been analyzed on proposed GMPLS optical network. The various algorithms involve Rivest Shamir Adleman (RSA) algorithm and Advanced Encryption Standard (AES) algorithm. The considered algorithms are very popular but have not been implemented on GMPLS optical networks in the literature which shows the novelty of the presented work. The network performance is compared for these algorithms in terms of various parameters like blocking probability and latency. The results reveal that RSA reduces the blocking probability < 0.005 and latency < 0.007 ms. This shows that RSA gives better performance than AES algorithm and enhance the quality of service considering blocking probability, latency and overheads in GMPLS optical networks leading to enhanced data protection.

## Introduction

**Figure 1:**
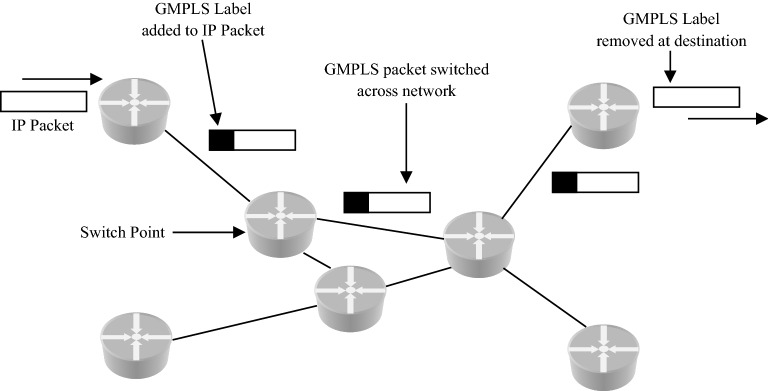
GMPLS Network.

The remarkable development of working clouds has fascinated and facilitated rigorous calculation on client devices with constrained resources. Primarily, well-groomed mobiles are allowed to organize information and computational exhaustive functions by influencing the required service representation of isolated data centers. Though, outsourcing private information to the distant data servers becomes difficult due to the cause of new concern related to information confidentiality and protection^1^. Protecting confidential data is a major worldwide challenge. Encryption algorithms play a key role in optical network protection systems. Conversely, these algorithms use a considerable quantity of computing resources^2^. The GMPLS optical networks are designed to survive IP's unreliable delivery. Various tunneling and Virtual Private Network (VPN) techniques are available so that GMPLS optical networks can work through IP clouds which help in restoration process. Also, the protocols in GMPLS optical networks apply security between the adjacent neighbors by using passwords. The solution to some problems like control plane security and IP addressing in GMPLS optical networks has been found to some extent by using Circuit-Switched End-to-End Transport Architecture (CHEETAH)^3^. The transfer of packets is connection leaning. They are routed along pre characterized Label Switched Paths (LSP) in GMPLS. It marks the traffic i.e. the packets have an identifier or a label to discriminate the LSPs. The Label Switched Router (LSR) applies the marked label to decide the LSP and then glance up the required LSP in its own forwarding table to be acquainted with the finest path to promote the packet and assigns a label to the subsequent hop. It employs information in the label to identify the packets and forwards them based on this label information rather than destination IP address in the packet^4^. The QoS in GMPLS network helps the device to handle the traffic efficiently as prescribed by each subscriber's network policy^5^. The GMPLS network is shown in Fig. 1.

**Figure 2:**
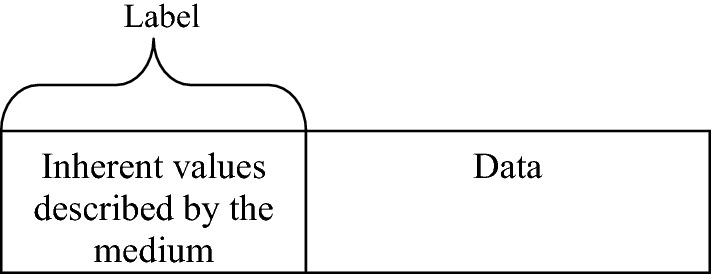
GMPLS packet format.

GMPLS provides enhancements to Multi Protocol Label Switched (MPLS) system to support set-up switching for wavelength, time and fiber taking packet switching also in account. The strategy utilizing GMPLS reduces the costs that engross bandwidth, switching and signaling^6,7^. The label comprises of the inherent values described by the medium employed in case of GMPLS network. No switch is required to interpret label in the legend of every packet. The label is an intrinsic component of the switch material and moreover, switching method rely on frequency, timeslot etc. as shown in packet format of GMPLS in Fig. 2. It makes use of different properties of material used by expected information flow to recognize the LSP which means that LSPs are inherently marked in GMPLS.

GMPLS enhances the system capability by allocating huge number of corresponding links between users in a system which is the fundamental necessity in optical communication where hundreds of similar paths may be present between a pair of users. It also makes possible switchovers to exchange channels, quick error recognition, error separation and reducing system downtime.

Till now, a number of popular data protection algorithms have been implemented on cloud computing networks and multimedia but not on GMPLS optical networks. The various data protection algorithms like AES, RSA, Blowfish, Data Encryption Standard (DES), Triple Data Encryption Standard (3DES), Two-parameter with a Wide-range system Mixed Coupled Map Lattice model (TWMCML), Double Parameters Fractal Sorting Matrix (DPFSM), Double Parameters Fractal Sorting Vector (DPFSV), Fractal Sorting Matrix (FSM), an algorithm based on the thought of improved ant colony walking path, triple-image encryption and hiding algorithm are implemented on cloud computing and image protection in the literature^2,8–15^. Moreover, the various encryption methods include the application of Quantum Key Distribution (QKD) for information security^16–19^. In literature, physical layer impairment (PLI)-aware shared path protection (SPP) scheme for single-link failures in transparent optical WDM mesh networks have been considered. PLI-aware integer linear programming (ILP) and heuristic algorithm for both SPP and dedicated-path protection (DPP) schemes have also been implemented. Their objectives were to maximize the network resource utilization and minimize the quality of transmission (QoT), blocking probability, restoration time, and computational time^20^.

We have extended the work by implementing AES and RSA on GMPLS optical networks and got good results considering blocking probability and latency which in turn increased data protection of the network. The RSA is Rivest Shamir Adleman algorithm in which the dissimilar keys are employed for encryption and decryption process. The algorithm is used for data protection and relies on modular exponentiation. In our previous papers, models for blocking probability have been developed^21^. Moreover, we have implemented Minimum Execution Time (MET) algorithm on our proposed GMPLS optical network for efficient bandwidth allocation in our conference paper^22^. Also, Weighted Round Robin (WRR) algorithm has been implemented on our proposed GMPLS optical network for well organized path computation in our second conference paper^23^. This paper extends our conference paper work and includes the implementation of different data protection algorithms individually and then the performance of all these algorithms is compared with each other. The previous papers were the basic ones so we presented in the conferences. This paper is meant for realistic scenario as far as future generation networks are concerned.

## GMPLS optical network

In the following segment, a plot that encloses the whole GMPLS optical network comprising various customers situated at their locations is offered. In addition, it illustrates the position of servers located in the arrangement.

**Figure 3:**
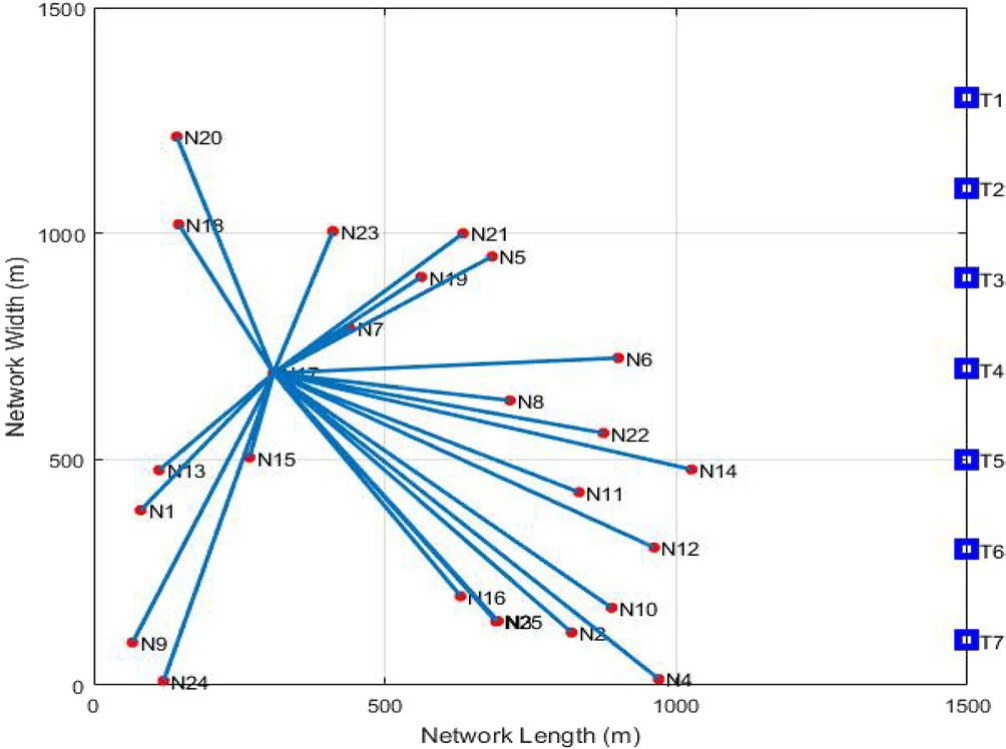
GMPLS optical network presenting generated requests.

The Fig. 3 is plotted for the GMPLS system including the clients and servers located at their specific places. Also, it depicts the requests arising from one user to the neighboring clients in the network. The red stars in the arrangement area represent the position of the users generating requests. The blue squares on the right side represent the position of servers performing the function of allocating wavelength and connection rates to demanding clients maintaining the routing table and connection constancy.

**Figure 4:**
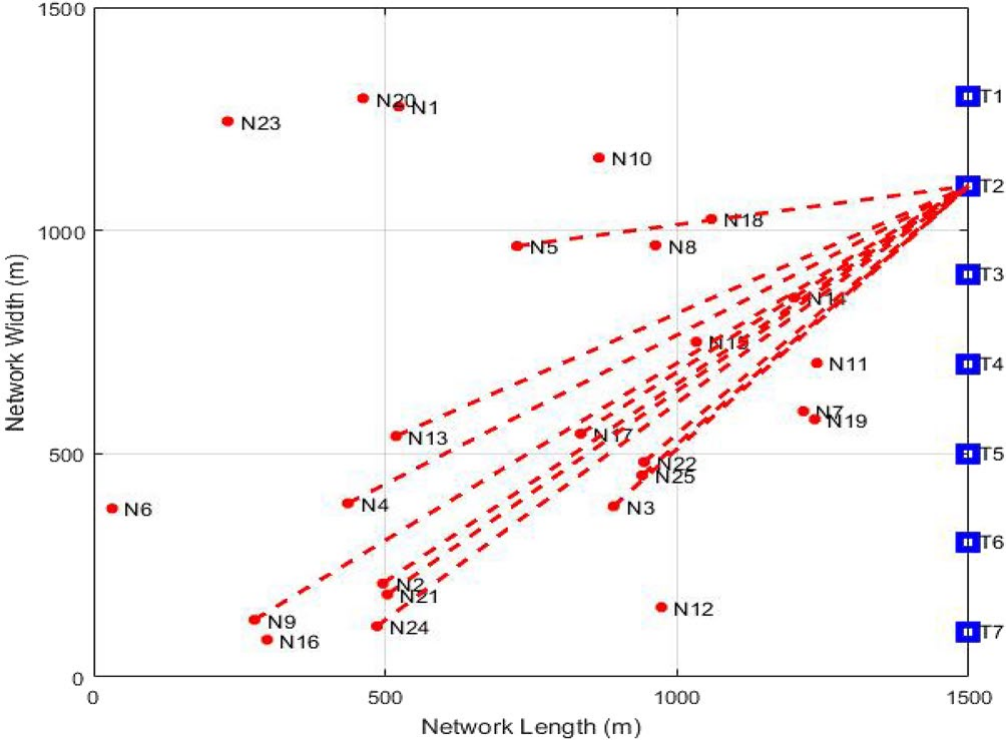
GMPLS optical network presenting link establishment.

The Fig. 4 corresponds to set-up of GMPLS optical network comprising of the clients and servers at their particular locations. It illustrates the allocation of server taking minimum time for task execution to the insisting nodes in optical system. At the time of request from the users to transfer information, the analysis of system servers is performed in terms of minimum execution time. This means that time taken by every server to execute that particular data transfer request is projected and then server taking minimum time to perform the task is allotted. Now, the chosen server distributes the bandwidth and connection rates to the clients and transfer of information happens. The red lines demonstrate the association between the nodes and the server. In this specific outcome, second server is discovered to perform the assignment at the earliest, therefore, is chosen for the link establishment.

## Simulation process

**Figure 5:**
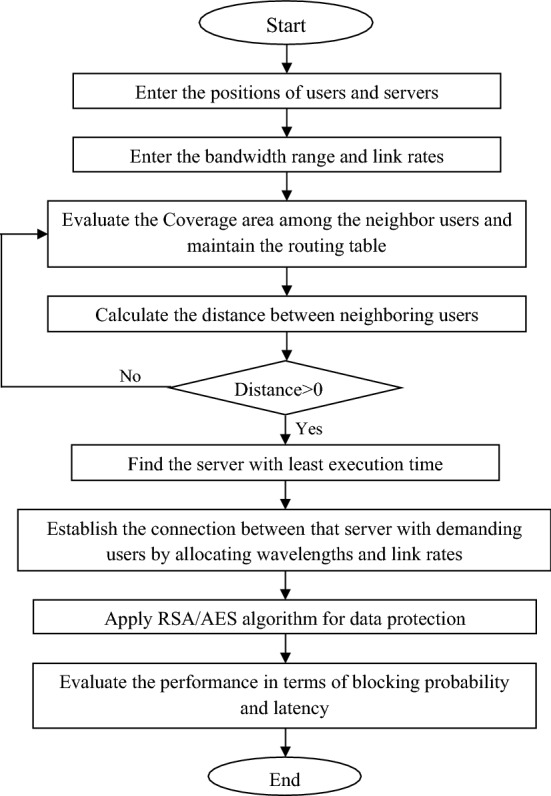
Flowchart for complete process.

Initially, the position of users and servers is defined in the specified network area. It is done dynamically as the users are not static in nature. The range of wavelengths and link rates are specified, keeping in mind the routing table and link stability. Then the coverage area among the neighboring users is calculated and the routing table is maintained. The flowchart of complete process is shown in Fig. 5.

The distance among all the users and server is investigated. If the distance is not greater than zero then it means the user is finding distance with itself so it needs to update the routing table. But if the distance is found to be greater than zero then the servers are analyzed for least time consumption to execute the task. Now, when the requests from different users occur, the time to execute the task by each server is estimated and the server having least predictable execution time period is allocated to the respective users. That is why the technique used is named as minimum execution time technique. After the allocation of servers to the users, the available wavelengths are assigned to the demanding users. The link rates are evaluated to maintain the link stabilities and are provided to the users. Then the data protection algorithms like RSA and AES are applied on the network to increase the information security in GMPLS optical network. Then the performance is evaluated in terms of blocking probability and latency in the GMPLS optical network.

## Data protection algorithms implemented on proposed GMPLS optical network

Nowadays, the data encryption developed to be very essential for the reason of handling the data transmission in all the fields. The large time consumed by an encryption system to encrypt the data leads to greater probability for the hackers to crack into the structure^24^. Internet of things illustrates the prototype of internetworking electronic devices to be associated to the internet and correspond with one another to trade and accumulate data via a wireless medium. These electronic procedures are outfitted with sensors, software, electronics and system connectivity that allow recognizing and communicating them with additional devices. But this leads to loss of confidential data to large extent. Thus, some encryption techniques are required to secure the data^25^. The two data protection algorithms: Rivest Shamir Adleman (RSA) algorithm and Advanced Encryption Standard algorithm are implemented on the proposed GMPLS optical network in this paper. The other data protection algorithms involve Data Encryption Standard (DES) algorithm, triple DES (3DES) algorithm and Blowfish algorithm^2^. One 64-bits key is employed in DES; 3DES utilizes three 64-bits keys whereas blowfish make use of different 32–448 bit keys^26,27^. However, several systems and attacks proved the drawbacks of DES and made it an apprehensive block cipher^28^. 3DES came like an enhancement of DES and has block length of 64 bits with key size of 192 bits. The encryption method is analogous to previous DES but pertained thrice to enhance the encryption intensity and average protected time. But a number of studies indicated that 3DES is more time-consuming than remaining block cipher schemes in terms of performance^28^. Blowfish takes a varying length key starting from 32 to 448 bits. It is an unpatented, license-free and is accessible by all clients for free. Blowfish has nearly deviations of 14 rounds^29^. The evaluation of TWMCML shows that the system has the characteristics of strong chaos, high sensitivity, broader parameter ranges and wider chaos range, which helps to enhance the security of chaotic sequences. It realizes double protection of private images under the premise of ensuring efficiency and safety. Security tests show that the application of TWMCML makes the encryption algorithm have a better ability to overcome conventional attacks^10^. DPFSM contains self-similar structures in the ordering of both elements and sub-blocks in the matrix. These two self-similar structures are determined by two different parameters. The proposal of DPFSM expands the fractal theory and solves the limitation of calculation accuracy on information security^11^. DPFSV has a good regulatory vector length and increases the diversity of information location change laws. The spatiotemporal chaotic system based on DPFSV exhibits better dynamics than that of the coupled map lattice (CML) system, verified by comparing their Kolmogorov–Sinai entropy, bifurcation diagram, information entropy, and mutual information^12^. The FSM is irregular, self-similar and infinitely iterative. Notably, scrambling images or information based on cluster of matrices can effectively improve encryption algorithm security^13^. In algorithm based on the thought of improved ant colony walking path, the image is scrambled by the row and column indexes and then the image is converted into one-dimensional array, and randomly scrambled^14^. In triple-image encryption and hiding algorithm, three grayscale images are simultaneously encrypted and embedded into a single color carrier image^15^. The literature review shows that AES and RSA perform better than all other above mentioned algorithms^30^. Thus, the network performance is analyzed considering blocking probability, latency and overheads for RSA and AES algorithms individually. They are implemented on the proposed GMPLS optical network by providing the same scenario which includes the bandwidth allocation using MET algorithm, path computation by WRR technique and the overheads are optimized using PSO as done in our earlier work^23^. After analyzing the performance individually, both the data protection algorithms are compared with each other in terms of blocking probability and latency.

### Rivest Shamir Adleman algorithm

**Figure 6:**
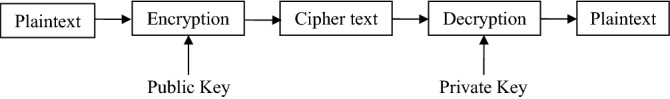
Block diagram for RSA algorithm.

The RSA cryptosystem is asymmetric in nature and depends on the spectacular distinction involving the simplicity of discovering big primes and complexity of factoring the multiplication of two big prime numbers. In this algorithm, the dissimilar keys are employed for encryption and decryption process. This reveals that A can be encrypted with e and decrypted by means of d. On the contrary, encryption can be performed by means of d and decryption with e. The duo of numbers (*n*, *e*) define the public key and can be available. The duo of numbers (*d*, *n*) is identified as the private key and needs to be kept secret. The number e is recognized as the public exponent, d is acknowledged as the private exponent and n is identified as the modulus^31,32^. The block diagram of RSA algorithm process is given in Fig. 6.

An asymmetric algorithm employs dissimilar keys for encryption process and decryption process. Everybody having knowledge about the public key is able to apply it to generate encrypted communications, however, merely the possessor of the secret key is able to decrypt them. On the other hand, the holder of the secret key can encrypt communications which can be decrypted by anyone having access to the public key. Anyone effectively decrypting these communications can be confident that no more than the possessor of the secret key could have encrypted them. This actuality is the major advantage of this algorithm.

#### Principle and working

In RSA algorithm, the plain text is encrypted as cipher text with public key and decrypted reverse as plaintext at the receiver side using private key. Both the keys can interchange their work also.

For encryption at the sender side, the plain text is transformed into cipher text c by using^31^ Eq. (1).

1 $$ c = m^{e} mod n $$

where, m represents the message, e indicates the public exponent and n is identified as modulus.

**Figure 7:**
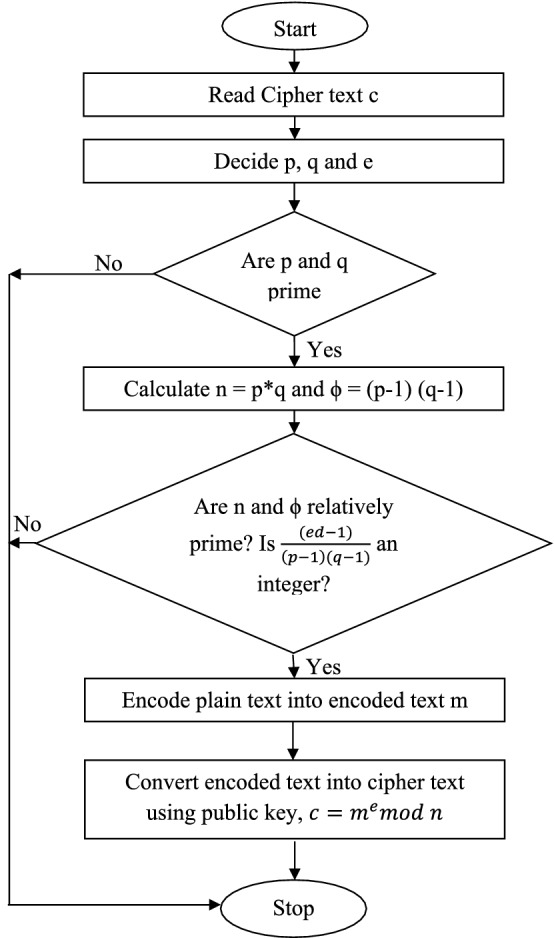
Flowchart for RSA encryption process.

The flowchart of encryption process for RSA algorithm is given in Fig. 7.

At the receiver side, the cipher text is again changed reverse to plain text by employing^31^ Eq. (2).

2 $$ m = c^{d} mod n $$

where d is private exponent.

**Figure 8:**
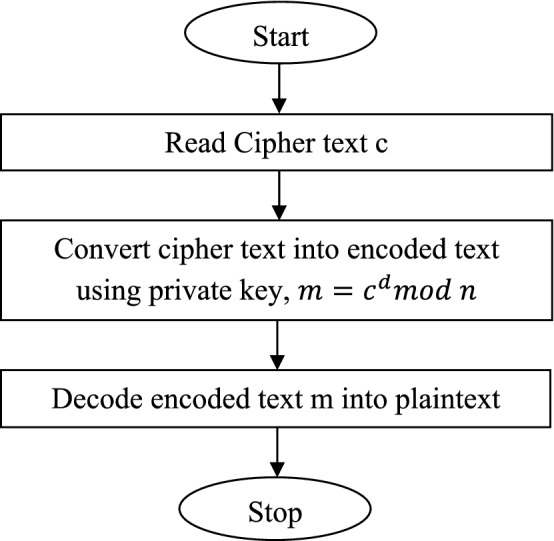
Flowchart for RSA decryption process.

The flowchart of decryption process for RSA algorithm is given in Fig. 8.

The process for RSA algorithm is explained with the help of an example stepwise as follows:
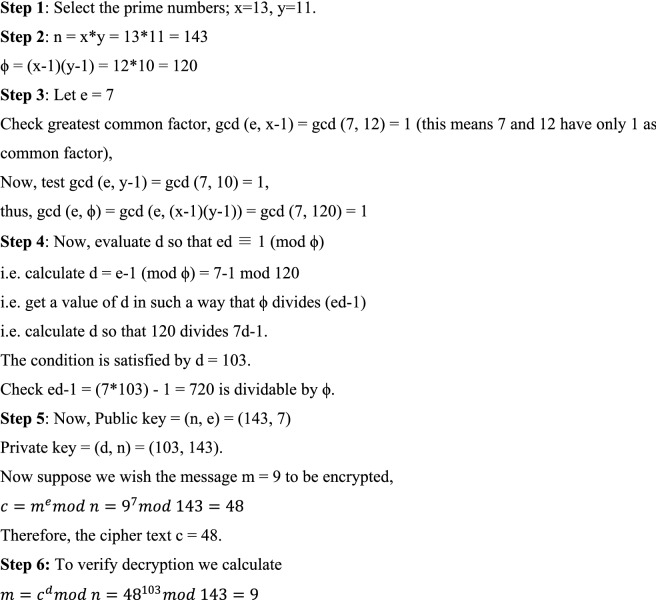


Here, in this example, we have taken small value of n to explain the process but in practice, big values for n are employed of the order of some hundred bits so that they cannot be predicted easily. This is because calculating c is simple but the opposite calculation of c is extremely complex for big values of n's in any case. The performance is assessed on the basis of blocking probability, latency and optimized overheads in the GMPLS optical network.

#### Results and discussions

In this section, the outcomes are plotted for blocking probability concerning the number of wavelengths accessible when demand from client occurs and traffic load. Then the graphs are plotted for different factors such as latency and overheads after implementing RSA algorithm.

**Figure 9:**
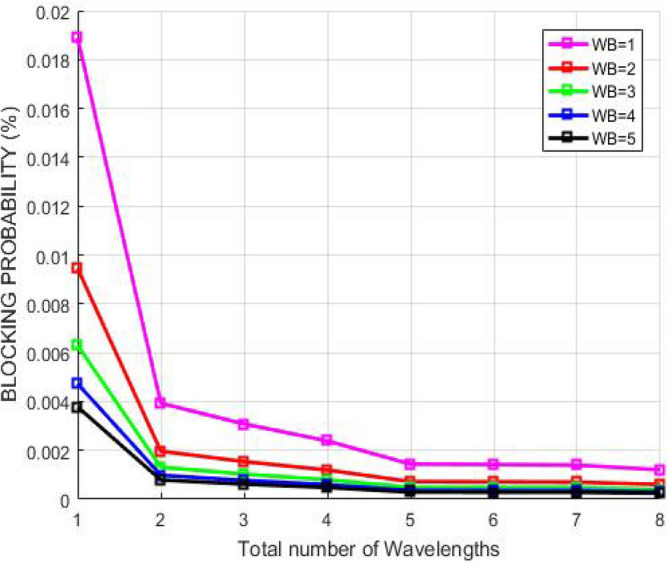
Blocking probability corresponding to number of accessible wavelengths.

Figure 9 illustrates the relation between the blocking probability and number of wavelengths accessible when demand from user occurs in diverse wavelength bands in GMPLS optical system. The blocking probability must be low so that there are fewer chances of call drops or request drops during busy hours. The anticipated method exploits the perception of smallest finishing time so wavelengths get vacant recurrently as the connection on the route gets adequate and shared bandwidths to transmit the packets from source to the destination user. Consequently, the number of offered wavelengths enhances resulting to decrease in the blocking probability. The plot shows the blocking probability reduces with increasing number of wavelengths which shows that wavelength conflicts are not occurring by using RSA. Thus, the data is neither being blocked nor detected by someone in the path. Therefore, the quality of service for the entire GMPLS system amplifies.

**Figure 10:**
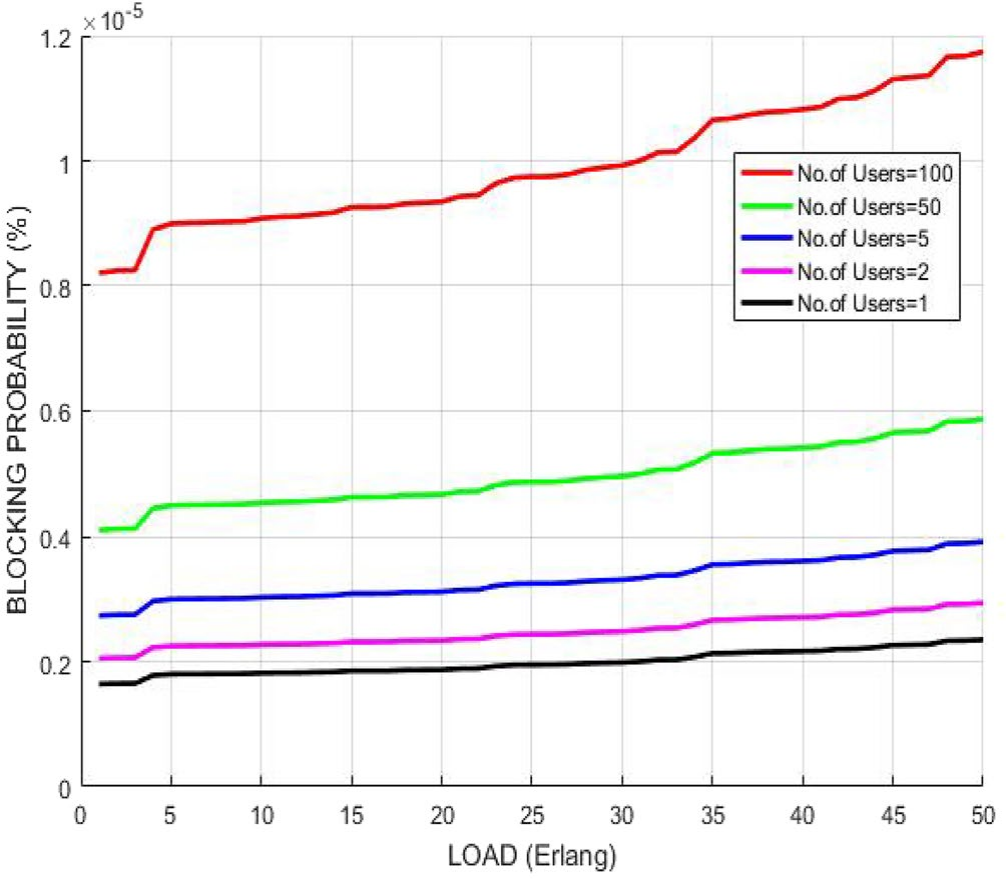
Blocking probability versus load.

The variation of blocking probability after implementing RSA with respect to the escalating traffic load in GMPLS optical arrangement is illustrated in Fig. 10. With the growing traffic load, channels or wavelengths get engaged resulting in blockage to user requests whereas network can handle large amount of traffic load with no blocking when the channels get vacant persistently due to the employment of smallest execution time model. The plot reveals that blocking probability rises with escalating traffic load, however, it is controlled one and its value remains below 1% which shows that it is tolerable to achieve high link stabilities.

**Figure 11:**
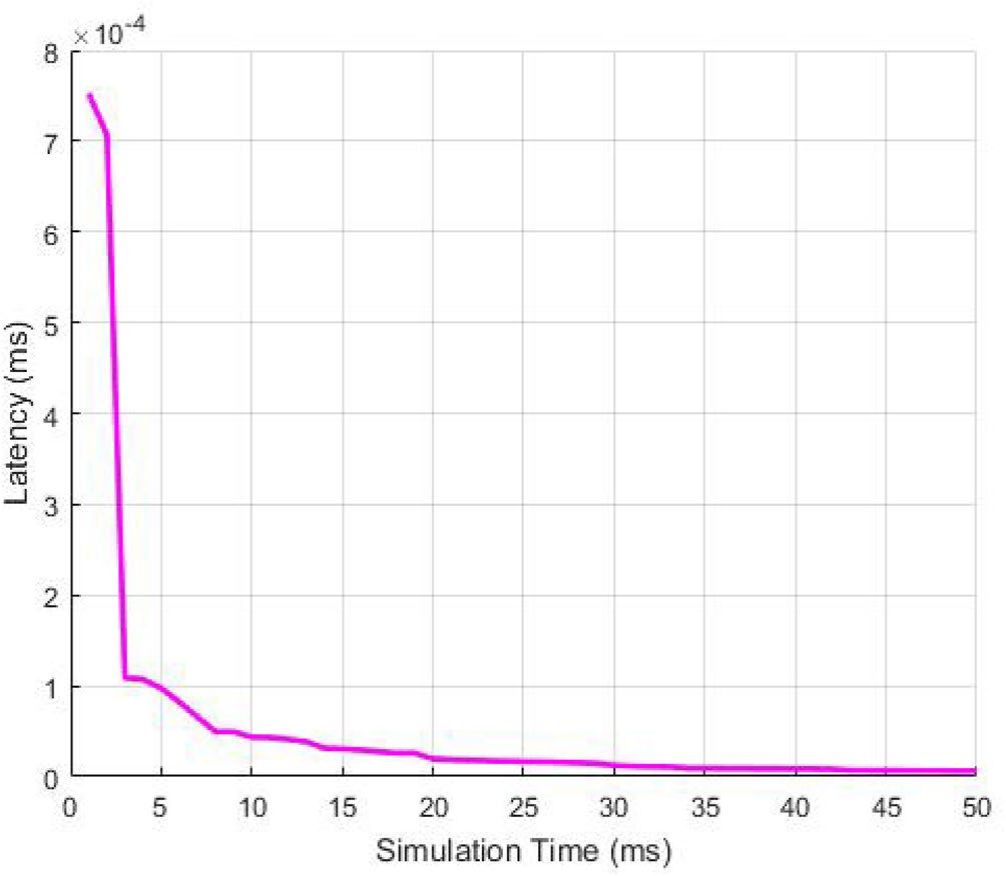
Latency as a function of Simulation Time.

Further, the latency in the transmission of data to the destination user from the source is plotted with respect to the simulation time in Fig. 11. Simulation time is defined as an approximate imitation of the process. The latency shows that how much time is required to deliver the packets to destination. If the latency is low then the computation time will also be less. The design indicates that the latency declines with rise in simulation time. The graph shows that the time taken to send out the data from the source to the destination user is very less even if the simulation time of the whole process is more by using RSA algorithm. Thus, RSA is able to achieve high packet deliveries and high throughput which in turn decreases the path delays and packet drops. Therefore, the network becomes more stable with high responses as the packet drops are less.

**Figure 12:**
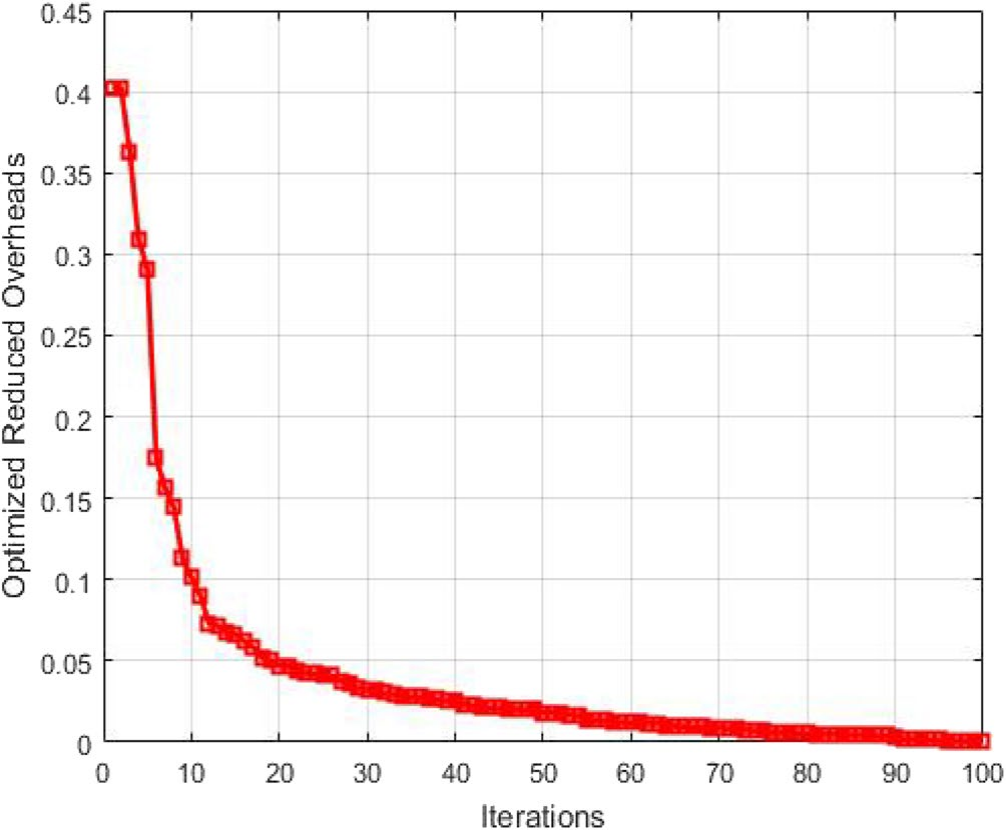
Optimized reduced overheads as a function of number of Iterations.

The overheads after optimization are plotted as a function of number of iterations in Fig. 12. The overheads are linked with loss of packets. The route overhead must be low for high packet deliveries and less packet losses. If the route overhead increases then the path delay increases and the load on the nodes through which packets are broadcasting also increases. So the route overhead must be low as much as possible. The plot reveals that as the number of iterations performed for optimization increases, the overheads get optimized resulting in the improvement of QoS in GMPLS optical networks.

### Advanced encryption standard algorithm

The AES algorithm is a symmetrical block cipher algorithm which acquires basic content in 128 bits sized blocks. It transforms these blocks into cipher content using keys of size 128, 192, and 256 bits. AES incorporates three block ciphers: AES-128, AES-192 and AES-256. Key size of 128 bits is incorporated in AES-128 to encrypt and decrypt a mass of communications whereas a key size of 192 bits is incorporated in AES-192 and a key size of 256 bits is incorporated in AES-256 to encrypt and decrypt communications^33^. The ciphers employ identical secret key for encryption and decryption process, thus, it becomes compulsory for both the dispatcher and the recipient to be acquainted with the confidential key and apply that key. The data can be categorized in three types: confidential, private and top secret. Any size of key can be applied to guard the confidential and private level but 128 bit key cannot be applied for top secret data, only 192 or 256-bit key is required. The complication for hackers in case of 256-bit key encryption extensively increases than in case of a 128-bit key. But the drawback of 256-bit key is that the processing power required is very large and it takes more time to accomplish. AES is utilized usually for defending information at rest like in case of self-encrypting disk drives. In contrast, RSA algorithm is frequently utilized in web browsers^31^. In case of symmetric process, same private key is used for transforming the plain content into cipher content and vice versa. Alternatively, two keys: public key and private key; are utilized in asymmetric process. If public key has been incorporated for encryption process then decryption process can be executed only with the associated private key and vice versa. RSA algorithm performs better for shielding the data transmission across geographic limits^34^. Conversely, AES keys are required to be secluded. The enhanced technique is to unite RSA encryption along with AES encryption in order to take advantage from the protection of RSA with the performance of AES. It is achieved by producing a momentary AES key and shielding it with RSA encryption process.

#### Principle and working

A Substitution-Permutation (SP) system is employed with several rounds in AES algorithm to generate cipher text. The key size decides the number of rounds to be involved. 10 rounds are incorporated for 128-bit key, 12 rounds for 192-bit key and 14 rounds for 258-bit key. Each round involve a round key but AES algorithm employ only one key for encryption and decryption so the expansion of key is required to obtain keys for every round including round 0^35^.

The following four steps are involved in every round of AES algorithm:

i. Byte substitution

Initially, the bytes of the block content need to be substituted depending on regulations ordered by predefined Substitution boxes (S-boxes). This process is also called Sub Bytes conversion. This step relies on nonlinear S-box to replace a byte in the position to a different byte.

ii. Rows shifting

Then permutation is done in this step. Here, each and every row is shifted by one except the first row. The major design at the back of this phase is to reallocate state bytes episodically to the left in every row except row zero. The bytes of row 0 stay unchanged and no permutation is incorporate for them. At the end of this phase, the size of resultant state is not altered and remains same as original 16 bytes but the arrangement of bytes is reallocated in the state. Only one byte is reallocated towards left circularly in the first row. The second row is modified by reallocating two bytes to the left. The last row involves shifting of three bytes towards left.

**Figure 13:**
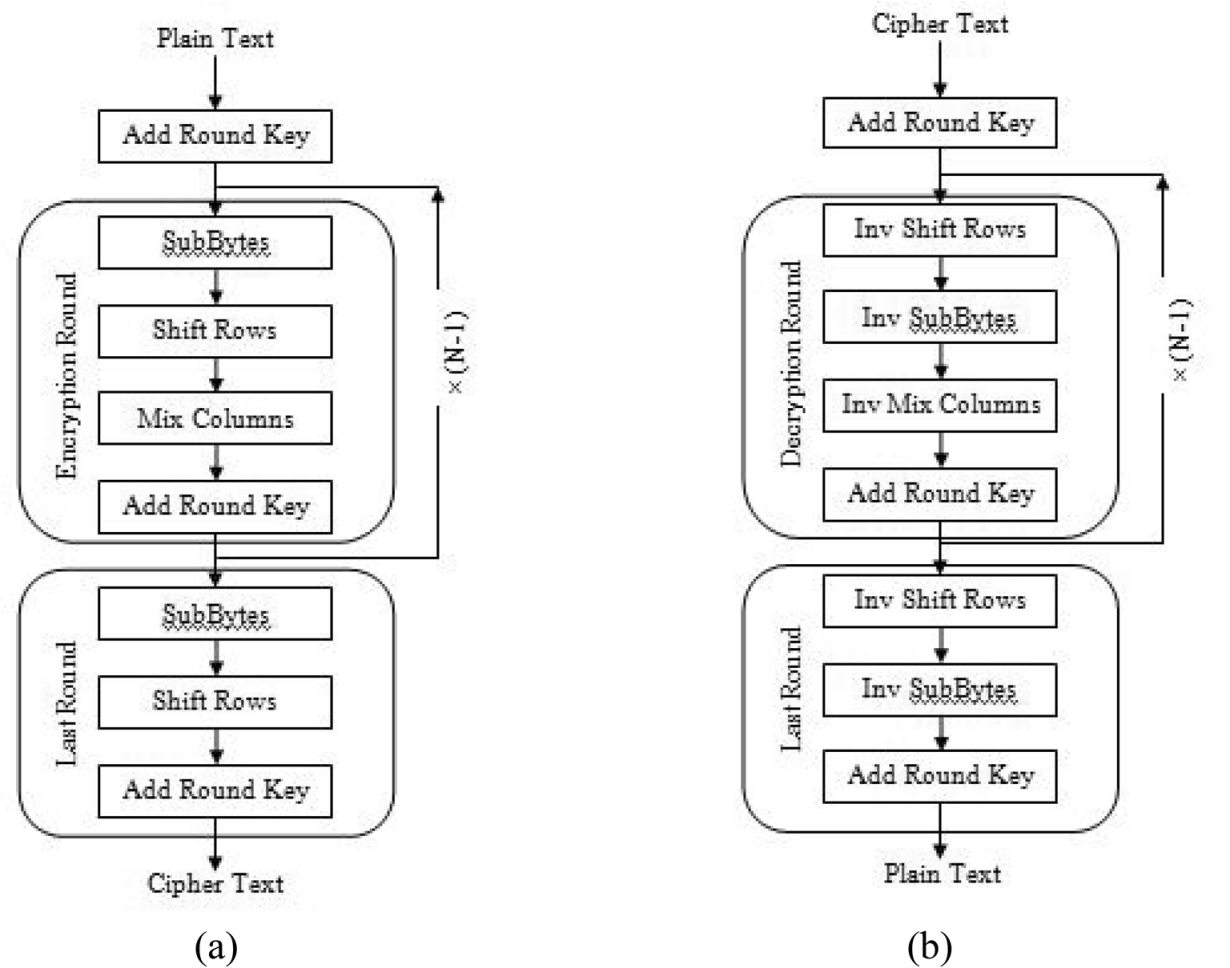
Flowchart for AES (**a**) encryption process (**b**) decryption process.

The flowchart for AES encryption and decryption are shown in Fig. 13.

iii. Column mixing

A further critical phase occurring in AES algorithm is mixing of columns. Now, the cipher is applied to muddle up the communication further by combination of block’s columns. This is done by carrying multiplication of the state. Each byte of one row in matrix transformation multiplies by each byte of the state (input data) column. The outcomes of these multiplications are exploited with XOR to generate fresh four bytes for the subsequent state.

iv. Round key addition

Finally, the information is XORed with the particular round key. The length of sub key and state remains alike. The sub key is added by joining every byte of the state with the subsequent byte of the sub key using bitwise XOR.

When these rounds are performed repetitively, these steps make sure that concluding cipher text is protected. Further, AES algorithm depends on expansion of key for encryption and decryption of data. A fresh key is incorporated in every round. The key expansion routine creates round keys word by word, where a word is an array of four bytes. The routine creates 4x (N + 1) words where N is the number of rounds.

#### Results and discussions

In the following segment, the consequences are plotted for blocking probability corresponding to the number of wavelengths vacant when demand from user arrives; and traffic load. The graphs are shown for diverse factors such as latency and overheads after implementing AES algorithm.

**Figure 14:**
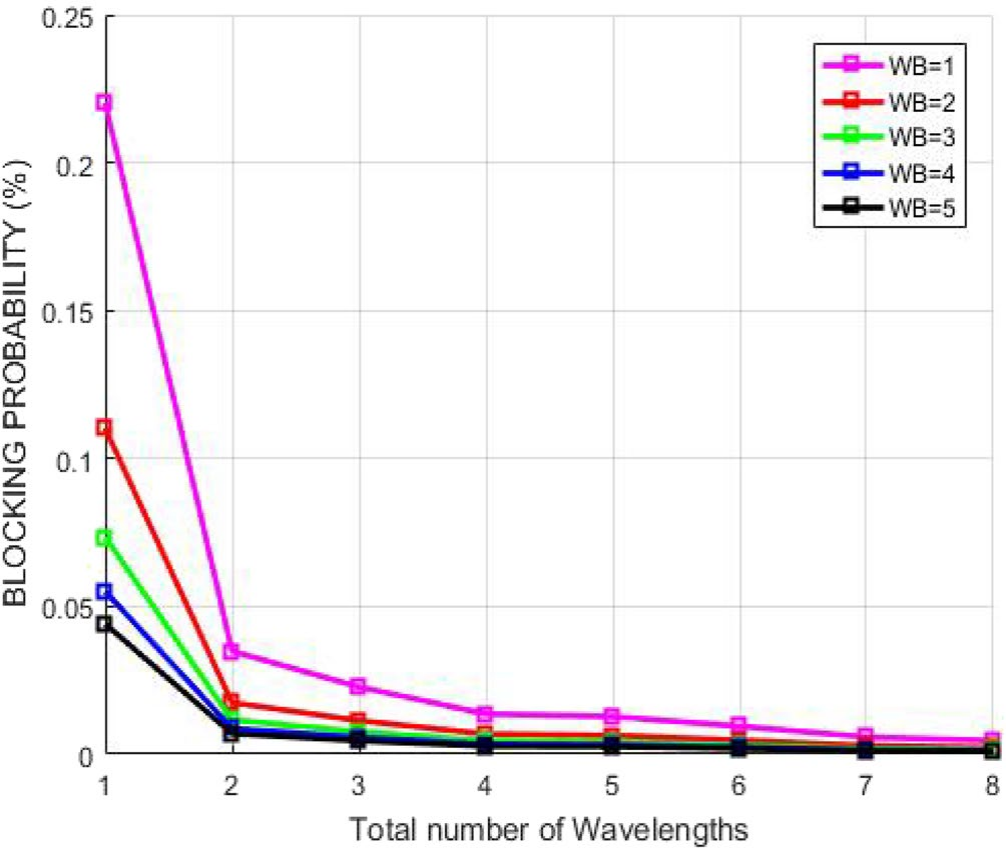
Blocking probability corresponding to number of accessible wavelengths.

Figure 14 describes the relation between the blocking probability and number of wavelengths accessible when demand from user occurs in diverse wavelength bands in GMPLS optical system. The blocking probability must be low so that there are fewer chances of call drops or request drops during busy hours. The anticipated method exploits the perception of smallest finishing time so wavelengths get vacant recurrently as the connection on the route gets adequate and shared bandwidths to transmit the packets from source to the destination user. Consequently, the number of offered wavelengths enhances resulting to decrease in the blocking probability. The plot shows the blocking probability reduces with increasing number of wavelengths which shows that wavelength conflicts are not occurring by using AES. Thus, the data is neither being blocked nor detected by someone in the path. Therefore, the quality of service for the complete GMPLS network amplifies.

**Figure 15:**
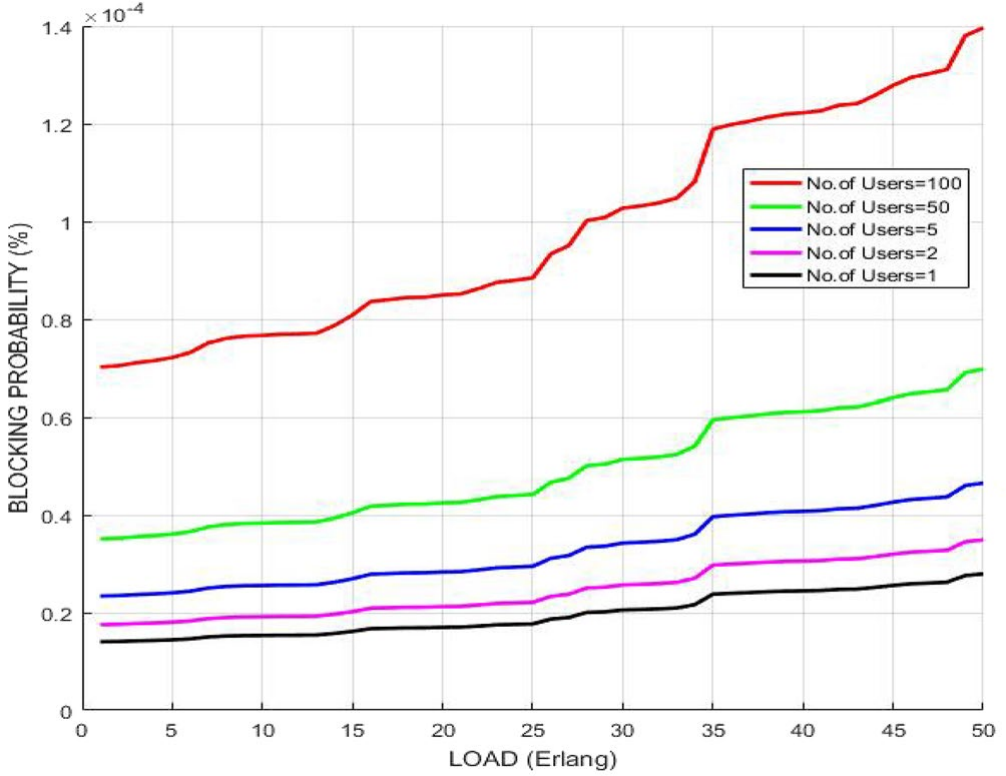
Blocking probability versus load.

The variation of blocking probability after implementing AES with respect to the escalating traffic load in GMPLS optical arrangement is illustrated in Fig. 15. With the growing traffic load, channels or wavelengths get engaged resulting in blockage to user requests whereas network can handle large amount of traffic load with no blocking when the channels get vacant persistently due to the employment of smallest execution time model. The plot reveals that blocking probability rises with escalating traffic load, however, it is controlled one and its value remains below 1% which shows that it is tolerable to achieve high link stabilities.

**Figure 16:**
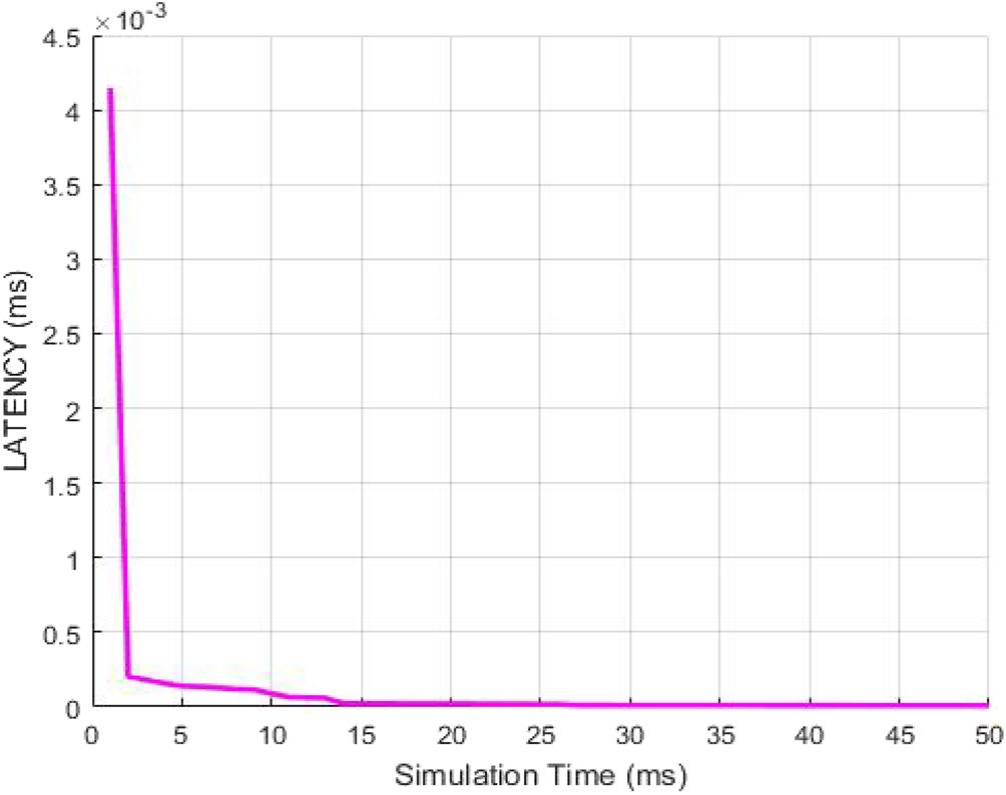
Latency as a function of Simulation Time.

Further, the latency in the transmission of data from the source to the destination user is plotted with respect to the simulation time in Fig. 16. Simulation time is defined as an approximate imitation of the process. The latency shows that how much time is required to deliver the packets to destination. If the latency is low then the computation time will also be less. The plot indicates that the latency reduces with rise in simulation time. The graph shows that the time taken to transmit the data from the source to the destination user is very less even if the simulation time of the whole process is more by using AES algorithm. Thus, AES is able to achieve high packet deliveries and high throughput which in turn decreases the path delays and packet drops. Therefore, the network becomes more stable with high responses as the packet drops are less.

**Figure 17:**
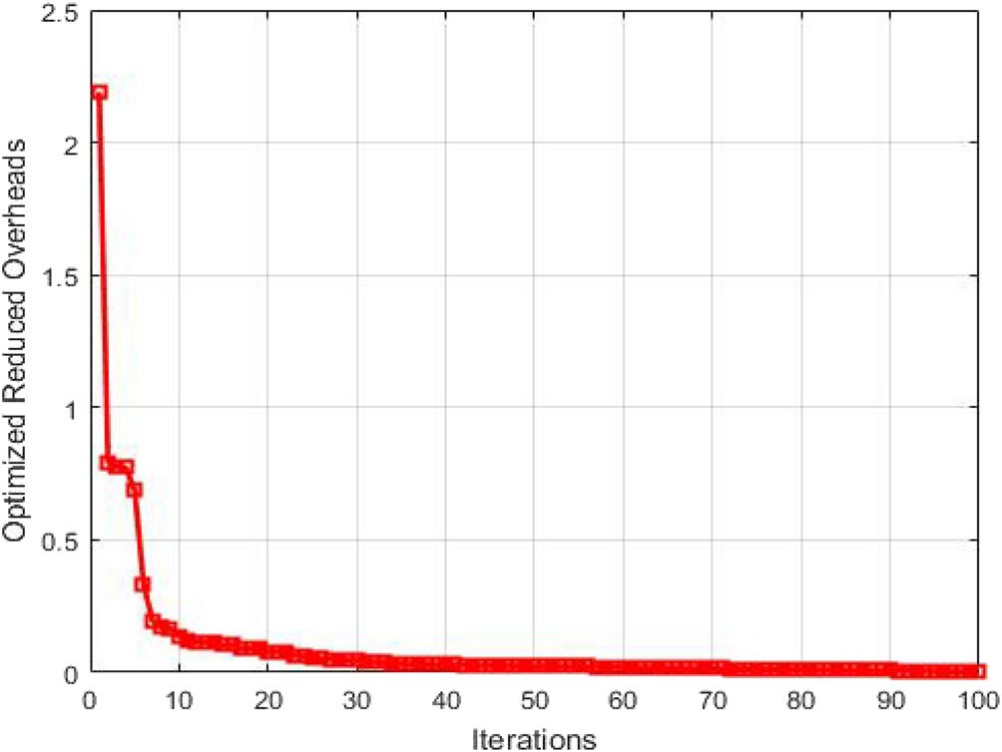
Optimized reduced overheads as a function of number of Iterations.

The overheads after optimization are plotted as a function of number of iterations in Fig. 17. The overheads are linked with loss of packets. The route overhead must be low for high packet deliveries and less packet losses. If the route overhead increases then the path delay increases and the load on the nodes through which packets are broadcasting also increases. So the route overhead must be low as much as possible. The plot reveals that as the number of iterations performed for optimization increases, the overheads get optimized resulting in the improvement of QoS in GMPLS optical networks.

## Comparison of different algorithms

In this section, the two algorithms discussed above are compared in terms of blocking probability and latency.

**Figure 18:**
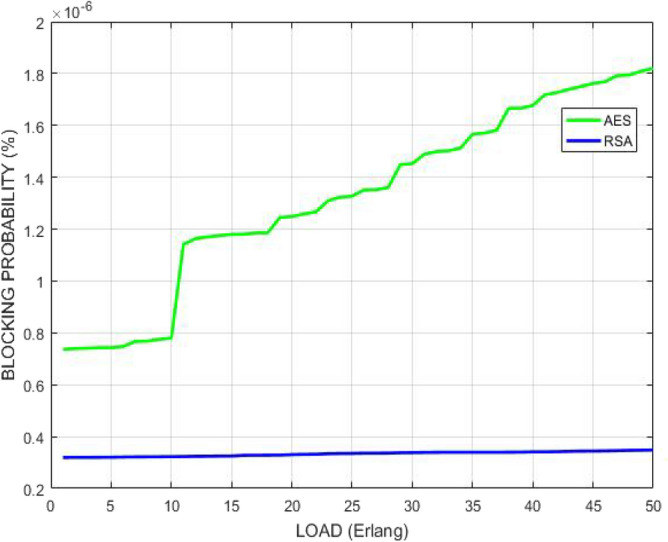
Blocking probability versus load.

Figure 18 compares the performance of GMPLS optical networks in terms of traffic load after implementing the above mentioned two different algorithms. The variation of blocking probability with increasing traffic load is plotted. The plot reveals that the RSA approach is able to achieve lower blocking probability with increasing traffic load than AES. This is because RSA uses the two different keys for encryption and decryption which provides more security to the network. The decreased blocking probability reveals the less number of wavelength continuity constraints and conflicts which in turn indicates that data is neither blocked nor detected in the communication channel.

**Figure 19:**
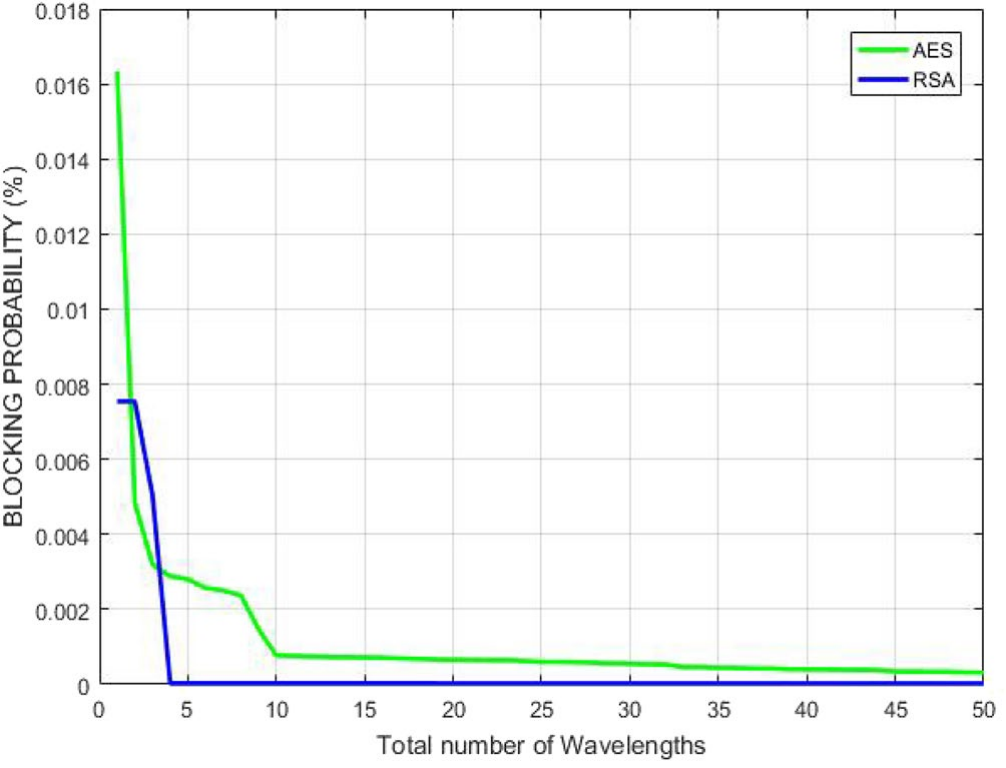
Blocking probability corresponding to number of offered wavelengths.

Figure 19 describes the correlation between blocking probability and number of wavelengths offered when demand from any user arrives in GMPLS optical system. The graph indicates that blocking probability lessens with rise in the number of available wavelengths. RSA is able to achieve lesser blocking probability than AES which shows that as the number of wavelengths increases, more traffic can be handled securely because the distinct channels can be allocated to the users. Thus, the probability of call block or drop decreases. Therefore, the quality of service for the complete network increases.

**Figure 20:**
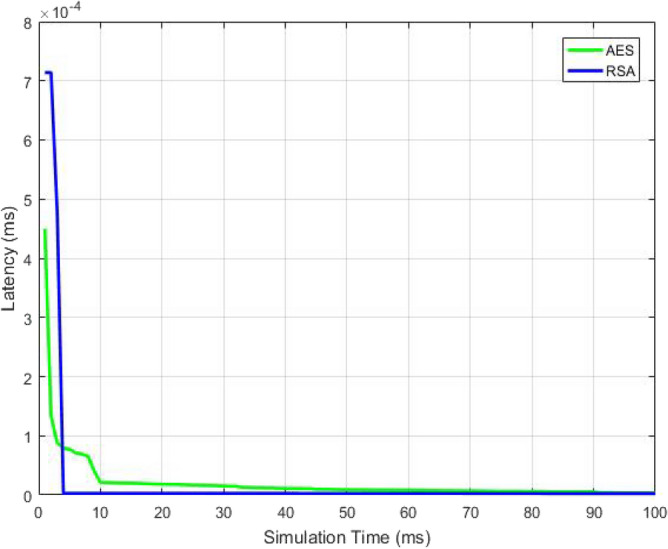
Latency as a function of Simulation Time.

The latency in the transference of information from dispatcher to the recipient is plotted with respect to the simulation time in Fig. 20. Simulation time is defined as an approximate imitation of the process. The plot indicates that latency declines with enhancing simulation time. The graph shows that the time taken for data transmission from the source to the destination user is less even if the simulation time of the whole process increases by using the RSA algorithm.

## Data protection aspects on proposed network

The routing and wavelength assignment process in optical network encounters a major problem of wavelength continuity constraint. In case of wavelength continuity constraint, the wavelengths must be assigned in such a manner that no two light paths sharing a single physical link use the same wavelength on that particular link. Moreover, in networks without the wavelength converters, the same wavelength must be used on all links of the light path. If the data is not secured during the transmission process, the wavelength continuity breaks and wavelength conflicts occur in the system leading to increased blocking probability^8^. Further, if the network latency is less, the time taken to receive next task decreases due to which blocking probability decreases which signifies that data is neither being blocked nor is detected by someone during transmission^9^. Thus, the decrease in blocking probability and latency of the network indicates the protection of data in the optical network.

## Conclusion

In this paper, GMPLS optical network is proposed which includes users with vibrant positions. Then different data protection algorithms are implemented on the proposed GMPLS optical network. The various algorithms implemented are Rivest Shamir Adleman algorithm and Advanced Encryption Standard algorithm. The above algorithms are considered due to their popularity and performance in various networks. Each algorithm is studied and compared on the proposed GMPLS optical network in terms of various parameters like blocking probability, latency and optimized reduced overheads. The results reveal that RSA gives the best performance among these algorithms and enhance the quality of service in GMPLS optical arrangements. The importance of our work is that these algorithms have not been implemented on GMPLS optical networks earlier as per the literature. The work can be extended by analyzing more algorithms like chaotic encryption methods and algorithms involving QKD on GMPLS optical networks and improving the QoS of the whole process.

## Data Availability

All data generated or analysed during this study are included in this published article.
